# Effectiveness of support from community health workers on the sustained use of a wearable monitoring device among community-dwelling older adults: A randomized trial protocol

**DOI:** 10.1371/journal.pone.0294517

**Published:** 2023-12-22

**Authors:** Arkers Kwan Ching Wong, Wai Chun Tso, Jing Jing Su, Vivian Chi Ching Hui, Karen Kit Sum Chow, Siu Man Wong, Bonnie Bo Wong, Frances Kam Yuet Wong

**Affiliations:** 1 School of Nursing, The Hong Kong Polytechnic University, Hung Hom, Hong Kong; 2 Elderly Center Division, Hong Kong Lutheran Social Service, Ho Man Tin, Hong Kong; Kurume University School of Medicine, JAPAN

## Abstract

**Background:**

Wearable monitoring devices, such as smartwatches and fitness bands, are health technologies for enhancing self-care management among community-dwelling older adults. While the evidence suggests that these devices can promote health, older adults often struggle to use them over the long term. Community health workers can effectively motivate older adults to change their health behaviors. This study proposes an intervention involving community health workers as peer supporters to promote sustained daily use of wearable monitoring devices among community-dwelling older adults.

**Methods:**

The intervention group in this randomized controlled trial will receive the Live with Wearable Monitoring Device program from trained community health workers with the support of a nurse and social workers through a one-time home visit and regular phone calls. The control group will receive only the wearable monitoring device. Data will be collected at baseline, 1 month, 3 months, and 6 months.

**Discussion:**

Merely providing older adults with wearable monitoring devices may not lead to the realization of the potential health benefits of these devices, as long-term usage can be challenging. The results of this trial can provide evidence for a new approach to enhancing self-management and community healthcare among community-dwelling older adults, ultimately improving their health outcomes.

**Impact:**

Wearable monitoring devices not only enable real-time monitoring of vital signs, but can also support tailored messaging and facilitate virtual communication between users and healthcare professionals. Despite considerable health benefits, there is evidence showing that older adults largely stop using them after a few months. This study is the first to use a peer support approach to help older adults incorporate a wearable monitoring device in their daily routines in conjunction with goal setting and regular reminders. This will boost the self-care ability of the older adults, allowing them to continue physically functioning in the community.

**Trial registration:**

This study was prospectively registered at clinicaltrials.gov (identifier: NCT05269303). Registration date: 24/2/2022.

## Introduction

Aging populations are a growing global phenomenon. There are currently about 1 billion people above the age of 60, a figure that is projected to increase to more than 2 billion, or about 22% of the total population, by 2050. Older adults commonly have chronic health conditions such as hypertension and diabetes, and require long term self-care management to prevent hospitalization and maintain their ability to live in the community [1]. There is promising evidence to show that self-care management strategies such as education [2] and self-monitoring [3] can promote better health outcomes among older adults. These self-care management strategies are now widely achievable using wearable monitoring devices (WMDs). WMD is a type of digital health technology that can be used for remote monitoring and has the potential to empower older adults in managing their self-care behaviors and improving their physiological and psychosocial health through early detection and prevention, while allowing them to live at ease in their own community.

WMDs such as fitness bands and smartwatches, which can be worn on the wrist, have sensors capable of detecting and recording physiological health data such as heart rate, step numbers, and the duration and intensity of an individual’s physical activity [4–6]. The data that are collected can be transmitted through a wireless sensor network to the server of a service provider for automatic processing, to provide immediate feedback to the user. The primary doctors and nurses of the users could also monitor the health data in real time and provide immediate, individualized feedback to the users. [7]. The evidence has shown that the use of WMDs can increase disease self-efficacy [8], enhance physical performance in daily activities [9], lower the incidence of falls [10], and increase physical activity [11] among older adults with chronic diseases.

Despite the benefits, recent reports have found that older adults have difficulty maintaining the long-term use of WMD. A study investigating the attitudes of older adults towards WMD has shown that 60% of participants stopped using the device after two weeks [12]. Another study aimed at investigating the effectiveness of WMD on the frequency of physical exercise showed that more than 20% of the older participants stopped using the WMD during the 12-week intervention period and another 50% discontinued the use of device 6 weeks after the intervention [13]. In fact, barriers such as the absence of clear instructions, knowledge, and support on handling the device, and a lack of motivation in learning new things have prevented older adults from continuing the use of WMDs [14].

Community Health Workers (CHW) have been recognized as paid workers who can motivate and support older adults in adhering to health-promoting and self-management activities. A qualitative study revealed that CHWs played a crucial role in facilitating the monitoring of vital signs, promoting physical exercise, and providing health education on older adults with chronic diseases [15]. In fact, CHWs do not necessarily have a professional medical or psychosocial background but do have commonalities with the person they are providing care to, such as age, socioeconomic background, and living place, which allow them to easily build strong rapport and connection with the care recipient [16]. In one study, older adults commented that they felt a sense of understanding, caring, and companionship from their CHW. They were more resilient to set-backs and more motivated to learn because their CHW was able to provide nonjudgmental support [17]. Previous studies discovered that a CHW-led program not only has significant effects on improving health outcomes for older adults [18], but is also cost-effective [19].

Compared with healthcare providers, CHWs are more familiar with the older adults and have more free time to teach them how to use the health features of the WMD and to overcome technical difficulties. There is, however, no study evaluating the effectiveness of CHW-led intervention programs on the sustained use of technological devices, including WMD, among community-dwelling older adults. In a pilot study in India, a training program was designed for CHWs to promote the use of WMDs to the public, but there was no data on the rates of usage and adherence [20]. The Live With Wearable Monitoring Device (LWMD) program is the first to integrate a peer support model to motivate older adults to sustain their use of a WMD for health monitoring and self-care management. This new approach has the potential to encourage the older adults to prolong and sustain their use of the device, thereby improving their long-term health and quality of life, and reducing their use of tertiary healthcare services.

There are three objectives in this study:

1. Assess the usability of the WMD from the perspective of older adults, including facilitators and barriers to the use of the device, level of satisfaction with the safety, convenience, and features of the device, attitude towards the use of the device, the perceived usefulness of the device, its perceived ease of use, and self-efficacy and level of anxiety in using the device.
2. Evaluate the feasibility of the Live With Wearable Monitoring Device program, including the recruitment rate, attrition rate, adverse events, and technical difficulties.
3. Identify the preliminary effectiveness of the LWMD program among older adults dwelling in the community, including their quality of life, utilization of health services, sustained use intention, and rate of adherence.

### Conceptual framework

This study was designed with reference to the Self-Determination Theory (SDT) [21]. According to this theory, people are more likely to adopt a particular behavior if they have an intrinsic motivation to carry out that behavior. Intrinsic motivation is influenced by three factors: autonomy, competence, and relatedness. Autonomy represents a sense of control over the behavior. Competence represents the perceived feeling of mastery of the behavior. Relatedness represents a feeling of being socially connected with people who share the same interest in the behavior. This study will provide options for older adults to integrate WMD into their daily life, educate them on the necessary knowledge and skills to operate the WMD, as well as strengthen the social connection and support from the CHWs.

## Research plan and methodology

### Study design

**Fig 1:**
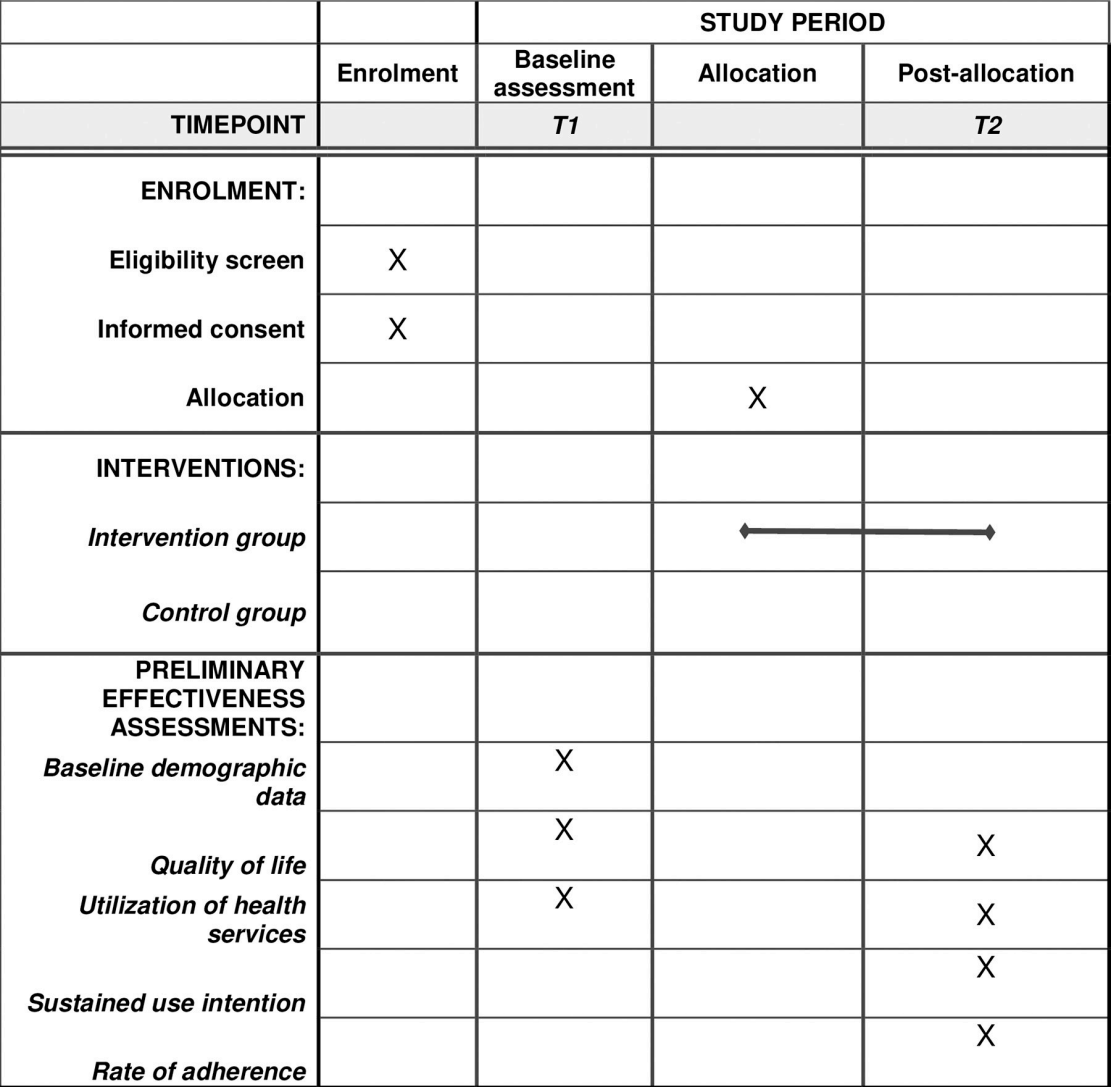
The schedule of enrolment, interventions, and assessments.

A randomized controlled trial design will be adopted in this study. The SPIRIT statement was used as a guideline for this protocol paper (Fig 1).

### Study setting, subjects, and recruitment strategies

The recruitment will be carried out in five community centers. Members of the community centers who have shown an interest in this program will be screened and recruited. The inclusion criteria are: (1) 60 years old or older, (2) owning a smartphone, (3) able to communicate in Cantonese or Mandarin, and (4) able to access the internet. The exclusion criteria are: (1) having a medically diagnosed cognitive impairment, (2) being bedbound, (3) already own a WMD, and (4) involved in other WMD-related studies.

A research assistant will explain details of the study to the eligible subjects. The written consent of the participants and their baseline data will be collected at the community centers. The participants will be randomly assigned to either the intervention or control group using group assignments generated by the Research Randomizer software. The group assignments will be sealed and opened sequentially by the principal investigator at the time of randomization. To maximize the level of double-blinding, participants will be informed that the intervention aimed to promote the use of the WMDs but will not be informed about their specific assignment to the intervention or control group. Furthermore, the research assistant responsible for data collection will remain blinded to the group allocation, while the providers, including community center staff, will not be blinded.

### Intervention

All subjects, regardless of whether they have been allocated to the intervention or control group, will be given a package that contains a WMD called ProVista Care®, a prepaid SIM card, a blood pressure monitor, and a pulse oximeter.

The WMD offers features that include vital signs monitoring, uploading, and recording, fall detection, location tracking, activity recording, medication and medical follow-up reminders, and phone calling. The subjects can upload their vital signs to the server of ProVista Care® and its smartphone application after measuring them using a blood pressure monitor and pulse oximeter. If abnormalities are detected, a research nurse in our program will follow up with the subjects via phone, based on protocols that have been developed in accordance with the guidelines of the National Institute for Health and Care Excellence. When deemed necessary, the nurse will make referrals to the emergency department, outpatient department, or hospital, based on their professional judgment and the established referral criteria.

All CHWs and subjects will receive a 60-minute training session on the use of the WMD, blood pressure monitor, pulse oximeter, and ProVista Care® app. The research team will also provide technical support throughout the study by leaving a hotline number in the community center.

#### Intervention group

**Fig 2:**
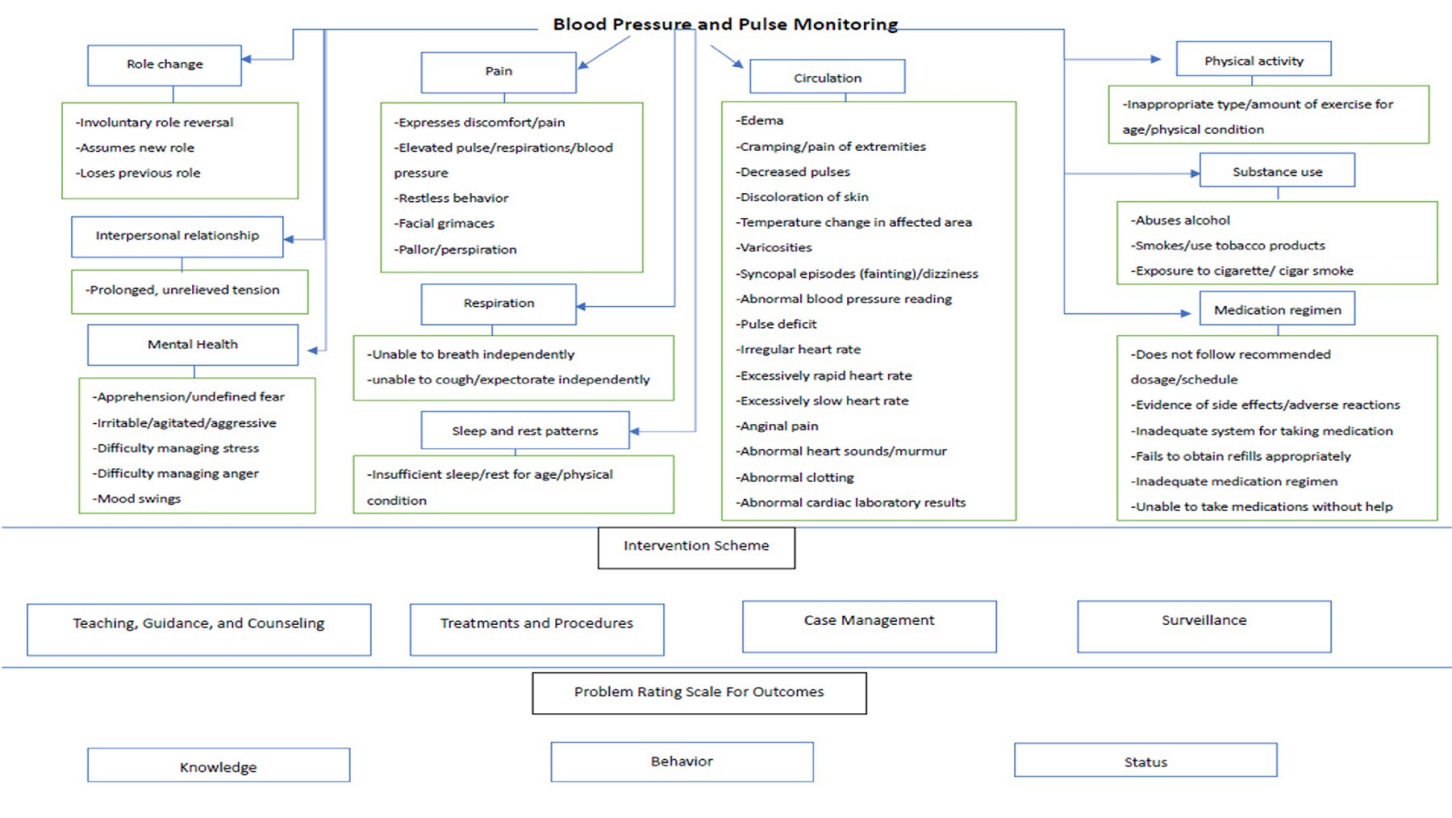
The problems of the Omaha System that linked with the blood pressure and pulse monitoring feature of the WMD.

The subjects in the intervention group will receive a three-month program called Live With Wearable Monitoring Device (LWMD), which will be conducted by CHWs under the supervision of our research nurse. The nurse and the trained CHWs will provide a first home visit session to subjects in the first month. The nurse will use the Omaha System, an evidence-based and comprehensive tool widely used in community-based practice, to identify physical and psychosocial health problems among the subjects that could be prevented or resolved through the use of the WMD [22]. For instance, the nurse will assess ten problem areas of the Omaha System, utilizing functions such as blood pressure and pulse monitoring in the WMD. These problem areas include role change, interpersonal relationships, mental health, pain, respiration, sleep and rest patterns, circulation, physical activity, substance use, and medication regimen (Fig 2).

In the event of abnormalities being detected, the nurse will suggest that participants utilize specific features of the WMD that are relevant to their identified health and social problems. They will provide recommendations regarding the duration and frequency of usage, as well as instructions on how to incorporate these features into their daily routines.

The CHWs will support the nurse with health assessments and the setting up of goals and action plans, and play an active role in communicating between the nurse and the subjects during the home visit. Starting from the third week, they will make regular phone calls to the subjects every other week until the twelfth week, to remind and encourage them to use the WMD. During the phone call, they will follow up on the problems that were identified by the nurse, on the subjects’ progress in attaining the goals, and suggest solutions for tackling the barriers preventing the subjects from implementing the action plans. The CHWs will seek help from the nurse when there is a need to modify the goals and action plan, or when any health issues arise that cannot be solved by the CHWs [23,24].

#### Control group

Subjects in the control group will use the WMD by themselves. They will not receive services from the CHWs and the research nurse unless abnormalities are detected by the WMD (e.g., abnormally high blood pressure), in which case the nurse will follow up on the subjects according to the protocols.

### Outcome measures

#### Usability

The aspect of the usability of the WMD includes facilitators and barriers to using the device, the level of satisfaction with the safety, convenience, and features of the device, attitude towards the use of the WMD, the perceived usefulness of the device and its perceived ease of use, and self-efficacy and level of anxiety in using the device. Subjects will be asked to take part in a focus group interview and provide feedback on the facilitators and barriers to using the device, and on their level of satisfaction with the safety, convenience, and features of the device. Attitudes on using the WMD, the perceived usefulness of the device and its perceived ease of use, self-efficacy, anxiety levels, and facilitating conditions in the use of the device will be measured using the senior technology acceptance model (STAM), which has been modified from a widely used and validated technology acceptance model. It measures using a 10-point Likert scale ranging from 1 (strongly disagree) to 10 (strongly agree). The higher the score, the better the outcome related to WMD usage. Anxiety level is the exception, where a higher score indicates greater anxiety [25,26].

#### Feasibility

The feasibility of this program will be evaluated by assessing the rate of recruitment, attrition rate, adverse events such as discomfort or injuries, and technical difficulties such as synchronization of the data from the WMD to the smartphone application. The data will be recorded by a designated staff member in each of the five centers. The rate of recruitment will be determined by dividing the number of eligible subjects who were recruited and randomized by the total number of eligible subjects. The rate of attrition will be calculated by dividing the number of subjects who quit or who could not be followed up by the total number of subjects who finished the program.

#### Preliminary effectiveness

The preliminary effectiveness outcomes are comprised of quality of life, utilization of health services, sustained use intention, and rate of adherence.

Quality of life will be evaluated using the Hong Kong version of the 5-level EuroQol 5-dimension scale (EQ-5D-5L), a validated descriptive system that has been used extensively in different jurisdictions. It consists of five dimensions: mobility, self-care, usual activities, pain or discomfort, and anxiety or depression. Each dimension has five levels of severity, namely, no problem, slight problem, moderate problem, severe problem, and extreme problem [27,28].

Utilization of health services will be measured by the number of attendances at a general practitioner’s office, emergency department, hospital, and general out-patient clinic. The data will be self-reported by subjects with confirmation via medical and attendance certificates [23].

The subjective primary outcome, sustained use intention, will be measured using a three-item, 5-point Likert scale measurement tool [29,30]. The scale ranges from 1 = strongly agree to 5 = strongly disagree. Lower scores indicate a higher chance of using the WMD sustainably.

The objective primary outcome, rate of adherence, will be evaluated by examining the number of days and the average length of time per day that the subject has worn the device. The data will be logged automatically into the system when the subject starts wearing the device.

Background demographics such as age, gender, marital status, educational level, primary caregiver, frequency of caretaking support, experience of using wearable monitoring devices, and eHealth literacy, will be collected at the baseline to account for differences between the groups.

### Data collection

The collection of data will occur at four time-points: baseline (T0), 1 month (T1), 3 months (T2), and 6 months (T3). T2 represents the immediate post-intervention period while T3 represents three months after the intervention for assessing the sustained effect of the program. At T0, baseline demographic data will be collected. Quantitative data will be collected at all four time-points through phone calls by the research assistant. Qualitative data will be collected at T2 through a semi-structured interview.

### Data analysis

Statistical tests will be performed using the software, Statistical Package for Social Sciences (SPSS) version 26. Descriptive analyses will be used to present the baseline demographic data. The differences and changes in outcomes for the quantitative data will be estimated using the Generalized Estimating Equation (GEE). For each outcome, (1) the between the intervention and control group (between-group) effects, (2) the within-group (time) effects, and (3) the group-by-time interaction (group X time) effects will be investigated. However, it is important to note that the validity of the GEE analysis relies on the assumption that data are missing completely at random (MCAR) [31]. Therefore, we will employ Little’s test to assess whether the missing data in our dataset are MCAR. If the test results are significant or indicate non-MCAR data, we will utilize Weighted Generalized Estimating Equation (WGEE) to analyze the outcome measures instead. Intention-to-treat will be our primary choice to analyze the data [32]. The level of significance will be set at p < .05 for a two-tailed test.

The qualitative data from the semi-structured interviews will be examined using the principles of deductive thematic analysis [33]. All focus group interviews will be conducted with audio recordings. Transcription and translation will be performed manually by research team members. To enhance conformability, an audit trail will be maintained to resolve any differences through agreement.

### Sample size

A power analysis was used to calculate the sample size for this study. We adopted usability as our primary outcome measure. To determine the effect size for our study, we referred to the Overall Acceptability Score [34], where the intervention group scored 45.2 (2.9) and the control group scored 40.4 (1.3). To account for potential differences in our local sample, which may consist of older individuals with lower mobile device literacy, we used a conservative approach and discounted the effect size by one-third for sample size calculation (which was 0.72). This was done to ensure an adequate sample size.

Assuming an alpha value of 0.05, a power of 80%, and an effect size of 0.72 [33], the sample size for each group should be 32. Accounting for a 10% drop-out rate, the total sample size in this study will be 70 (i.e., 35 per group).

### Ethical considerations

The study was approved by the Ethics Committee of the Hong Kong Polytechnic University (HSEARS20220429001). An information sheet and consent form will be given to the potential subjects during the recruitment process. A thorough explanation of the study will be given to ensure that the subjects are all well informed before their written informed consent is collected. Participation is completely voluntary, and they reserve the right to drop out at any time during the study. Only subjects who have signed the written consent form will be included in the study.

## Discussion

Amidst the COVID-19 pandemic and the implementation of preventive measures, the advancement, popularity, and acceptance of health technologies accelerated, particularly among community-dwelling older adults [35,36]. WMDs allow healthcare professionals to remotely monitor the health of community-dwelling older adults and provide immediate support when necessary. Despite their advantages, the evidence suggests that older adults tend to use WMDs only for a short period of time. Therefore, it is paramount to develop innovative approaches to enhance the motivation of older adults to use WMDs sustainably, so that the benefits of using them can be realized.

We have designed a novel approach that includes community health workers to enhance the sustainability of using WMDs among community-dwelling older adults. To the best of our knowledge, the proposed study is the first randomized controlled trial to evaluate the sustainability and effectiveness of WMDs with an additional intervention that includes community health workers as peer supporters. The study utilizes commercially available WMDs, which increases the real-world practicality of the study. In addition, the assessment tools adopted from prior empirical studies have been validated, increasing the reliability of the study. The control group, which does not involve CHWs, allows for a comparison to be made and provides insight into the significance of peer support. If successful, this study will generate evidence on interventions involving CHWs to enhance the long-term use of WMD in promoting the health of community-dwelling older adults. Moreover, by utilizing WMD technology and expanding the role of community health workers, the suggested intervention could overcome the geographic challenges to implementing community healthcare and become a cost-effective addition to ease the shortage of healthcare professionals.

Some limitations are anticipated. First, since the study will recruit members from five local community centers onsite, the potential subjects might be more socially active than those who do not often join activities organized by community centers, which could introduce a selection bias that could affect the results; therefore, the findings of this study need to be carefully examined. Second, the study was conducted with a group of Chinese-speaking older adults. Previous studies have shown differences between cultures in the adoption of WMD. The Chinese population is inclined to be more socially influenced to adopt the use of WMD compared to their Swiss counterparts [37]. Therefore, the peer support effect might be more profound in the Chinese culture than in some other cultures.

## Conclusion

Wearable monitoring devices (WMD), such as smartwatches, have shown promising health benefits, but maintaining long-term use among older adults remains a challenge. This article proposes a randomized controlled trial protocol that involves community health workers as peer supporters to increase the sustainability of using WMDs among community-dwelling older adults. If proven successful, older adults can enjoy the health benefits arising from the use of the WMD and be able to live independently in the community for longer than might overwise have been the case.

## Supporting information

S1 Checklist SPIRIT 2013 checklist: Recommended items to address in a clinical trial protocol and related documents*. (DOC)
